# Japan: Diagnosis and Management of Stevens-Johnson Syndrome/Toxic Epidermal Necrolysis With Severe Ocular Complications

**DOI:** 10.3389/fmed.2021.657327

**Published:** 2021-07-28

**Authors:** Chie Sotozono, Mayumi Ueta, Shigeru Kinoshita

**Affiliations:** ^1^Department of Ophthalmology, Kyoto Prefectural University of Medicine, Kyoto, Japan; ^2^Department of Frontier Medical Science and Technology for Ophthalmology, Kyoto Prefectural University of Medicine, Kyoto, Japan

**Keywords:** Stevens-Johnson syndrome, toxic epidermal necrolysis, cultivated oral mucosal epithelial transplantation, Limbal-Rigid contact lens, Japan

## Abstract

In 2005, the “Japanese Research Committee on Severe Cutaneous Adverse Reaction” (J-SCAR) presented the official “Diagnostic Criteria” for SJS/TEN, and the specific ocular findings are included in these very important criteria. In SJS/TEN cases involving ocular disorder, conjunctivitis often occurs prior to the onset of the high fever. In a Japanese survey, ocular involvement was observed in 77% of the cases, and the incidence of ocular sequelae increased depending on the score of the acute ocular severity findings. Pseudo-membrane formation and epithelial defects are considered to be high-risk signs of ocular sequelae. At the chronic stage, limbal stem cell deficiency, visual disturbance, and severe dryness of the ocular surface are the primary disease characteristics. In 2002, we started performing Cultivated Oral Mucosal Epithelial Transplantation (COMET) for the treatment of severe ocular disorders, including SJS/TEN. As an additional treatment method, we developed a new type of rigid contact lens (CL) that is 13 to 14.0-mm in diameter, known as the “Limbal Rigid Contact Lens (Limbal CL).” Our Limbal Rigid CL greatly enhances the postoperative outcome of COMET. The detection rate of ocular surface bacteria is high in SJS/TEN cases. Thus, appropriate use of topical antibiotics reduces the risk of ocular surface inflammation. Moreover, rebamipide is an ophthalmic solution for dry eye that was developed in Japan, and it also has the effect of suppressing ocular surface inflammation. From disease onset until the chronic stage, the control of inflammation and stem cell loss is key to successfully treating eyes afflicted with SJS/TEN.

## Introduction

Stevens-Johnson syndrome (SJS), and its more severe variant, toxic epidermal necrolysis (TEN), are acute systemic disorders that can affect anyone, and at any age (1–3). A variety of drugs can be the cause of SJS/TEN. At the onset of the disease, a definitive diagnosis of SJS and TEN is often complex and confusing.

In Japan, the “Japanese Research Committee on Severe Cutaneous Adverse Reaction” (J-SCAR) has been conducting diligent and extensive work on the diagnosis and treatment of SJS/TEN over the past two decades. In 2005, J-SCAR presented the official “Diagnostic Criteria and Systemic Severity Index Score” for SJS/TEN (4). Since then, SJS/TEN has been diagnosed based on these criteria. Importantly, the disease-specific ocular characteristics are now included as one of the supportive findings, and these criteria has enabled ophthalmologists to be a valued clinical “team member” for the diagnosis and treatment of SJS/TEN at the acute stage.

Both SJS and TEN are systemically self-limited lasting 6–8 weeks after onset. However, in SJS/TEN cases with severe ocular complications (SOC), persistent epithelial defects (PED) on the ocular surface can linger, ultimately resulting in ulceration and perforation (5). Finally, visual impairment and severe dryness of the eye remain as ocular sequelae (6, 7). At present, there is no standardized treatment strategy for SJS/TEN-related blindness worldwide. Patients with SJS or TEN require life-long management for ocular discomfort and morbidity. Our group has now developed both surgical and non-surgical therapeutic methods for the successful treatment of SJS/TEN.

In this review article, we describe the current diagnostic and therapeutic strategies for SJS/TEN with SOC now practiced in Japan, and provide a detailed summary of the multi-year clinical research and comprehensive Japanese survey on SJS/TEN conducted by Kyoto Prefectural University of Medicine, Kyoto, Japan.

## SJS/TEN With Ocular Involvement at the Acute Stage

At disease onset, ocular involvement in SJS/TEN is often easily overlooked due to the serious general symptoms and high lethality. Thus, the clinical characteristics of SJS/TEN with SOC need to be well-understood for early diagnosis and successful treatment.

### Characteristic Findings

**Figure 1:**
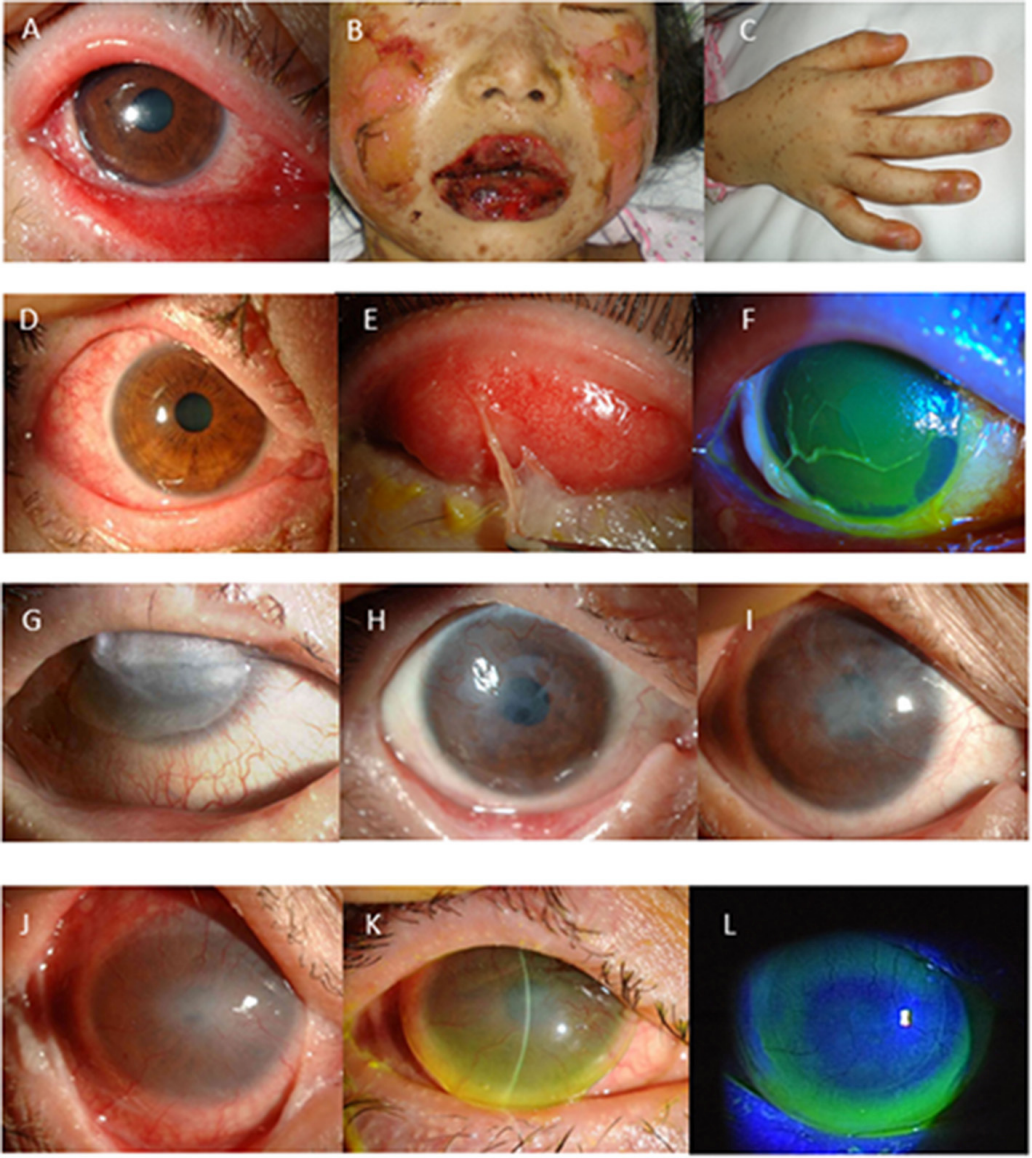
**(A–F)** Representative photographs showing Stevens-Johnson syndrome/toxic epidermal necrolysis (SJS/TEN) at disease onset with severe ocular complications (SOC). **(A)** Conjunctivitis, which was accompanied by extensive loss of corneal and conjunctival epithelium. **(B)** Swollen and crusted lips. **(C)** Paronychia. In 66% of the patients, conjunctival hyperemia **(D)** preceded skin eruption. Pseudomembrane formation **(E)** and corneal or conjunctival epithelial defect **(F)** develop at the acute stage. **(G–I)** Long-term effect of cultivated oral mucosal epithelial transplantation (COMET). Images of SJS/TEN cases obtained at before **(G)** and at 2-years **(H)** and 5-years postoperative **(I)**. **(J–L)** Use of a limbal-supported rigid-type contact lens (Limbal-Rigid CL; Sun Contact Lens) for the treatment of SJS/TEN with ocular disorders. Representative appearances of the eyes with SJS/TEN at before **(J)** and after 3-months use of the Limbal-Rigid CL **(K,L)**.

Our findings revealed that among 94 SJS/TEN patients with SOC, 75 (82%) experienced common cold-like symptoms (general malaise, slight fever, sore throat, etc.) that preceded the skin eruptions. In all patients, except one, the disease was accompanied by very high fever (above 39°C) at onset. Acute conjunctivitis and oral involvement (blisters, erosions, and bleeding of the mouth and lips) occurred in all patients who could recollect their symptoms in detail. Fingernail loss at the acute stage or deformation at presentation existed in all patients, thus suggesting that paronychia occurred in all patients at the acute phase (Figures 1A–C). Other mucous-membrane involvements included those of the pharynx, respiratory tract, or ear canal (8). Forty-two patients reported episodes of acute conjunctivitis from several hours to 4 days prior to the skin eruptions, and 21 patients reported that skin eruptions and conjunctivitis occurred simultaneously. Only 1 patient reported the occurrence of conjunctivitis post skin eruption. All patients reported remembering their eye symptoms such as bilateral red eye or ocular pain at disease onset. Surprisingly, 11 patients were diagnosed with acute conjunctivitis by ophthalmologists prior to the development of systemic eruptions. Thus, ophthalmologists should be aware that acute conjunctivitis can occur prior to skin eruptions (8).

### Acute Ocular Severity Score

Typically, pseudomembrane formation with corneal and/or conjunctival erosions occurs at the acute stage of SJS/TEN. Hence, we speculated that inflammation and epithelial defects are key aspects of the ocular findings at the acute stage and developed the “Acute Ocular Severity Score” on a scale from 0 to 3 (none, mild, severe, and very severe) according to the existence of hyperemia, corneal or conjunctival epithelial defect, and pseudomembrane formation (Figures 1D–F) (4). In a Japanese survey, ocular involvement was seen in 104 (77%) of 135 SJS/TEN cases (87 SJS and 48 TEN cases arising between 2005 and 2007), and pseudomembrane formation and/or epithelial defects were seen in 62 of those cases (46%) (4). The incidence of ocular sequelae increased depending on the score of the acute ocular severity. Thus, pseudomembrane formation and epithelial defects are considered to be high-risk signs of ocular sequelae.

As shown in our previous study, patient age and NSAIDs or cold remedies as the exposed drugs were the predictive factors for the increase of acute ocular severity (4) (Table 1). In our previously published retrospective studies on SJS/TEN cases at the chronic phase, mean patient age was between 20 and 30 years at disease onset and cold remedies were the exposed drugs that were used in more than 24% of the cases (7, 8) (Table 2).

**Table 1:** Univariate Logistic Regression Analysis Of The Association Between Variables At Onset And Acute Ocular Severity In The Patients with Stevens-Johnson syndrome (SJS) and toxic epidermal necrolysis (TEN) [modified version of the table presented in (4)].

**Variable at the onset *n* = 135**	**Univariate logistic regression**		
	**OR**	**95%CI**	***P*-value**
Disease: TEN (vs. SJS)	1.47	0.72–2.98	0.287
Gender: Male (vs. Female)	0.78	0.39–1.54	0.466
Age at the onset (years)	0.97	0.96–0.99	0.004
Age at onset (years): >50 (vs. 50 ≤)	0.36	0.18–0.72	0.004
NSAIDs	2.04	1.02–4.1	0.045
Cold-remedies	5.51	1.72–17.62	0.004
NSAIDs or cold-remedies	2.68	1.33–5.38	0.006
Antibiotics	0.66	0.27–1.63	0.363
Anticonvulsants	0.72	0.33–1.58	0.415

*OR, odds ratio; CI, confidence interval; NSAIDs, non-steroidal anti-inflammatory drugs*.

**Table 2:** Demographics of the Stevens-Johnson syndrome and toxic epidermal necrolysis cases seen at the acute and chronic phase.

		**Acute Phase Cases**				**Chronic Phase Cases**	
		**Acute Ocular Severity Score**				**Multi-center**	**KPUM**
		**0**	**1**	**2**	**3**		
No of cases		31	42	39	23	73	94
Disease category	SJS	19	31	22	15		
	TEN	12	11	17	8		
Age at onset (years)	SJS	57.1 ± 18.2	52.3 ± 18.7	54.4 ± 19.1	39.2 ± 19.2	28.4 ± 18.2	26.2 ± 18.8
Mean ± SD	TEN	62.0 ± 14.5	64.6 ± 25.5	47.9 ± 19.0	39.6 ± 14.6		
Duration of illness (years)						18.8 ± 15.5	16.1 ± 15.2
Mean ± SD			
**Exposed drug: No of cases (%)**							
NSAIDs	Yes	12 (38.7%)	12 (28.6%)	18 (46.2%)	13 (56.5%)	10 (13.7%)	19 (20.2%)
Cold-remedies	Yes	1 (3.2%)	3 (7.1%)	9 (23.1%)	6 (26.1%)	18 (24.7%)	30 (31.9%)
Antibiotics	Yes	4 (12.9%)	11 (26.2%)	7 (17.9%)	2 (8.7%)	21 (28.8%)	23 (24.5%)
Anticonvulsants	Yes	6 (19.4%)	15 (35.7%)	10 (25.6%)	4 (7.4%)	6 (8.2%)	5 (5.3%)

*SJS, Stevens-Johnson syndrome; TEN, toxic epidermal necrolysis; SD, standard deviation; NSAIDs, non-steroidal anti-inflammatory drugs*.

*The Acute Ocular Severity Score was defined as Score 0 (none) = no ocular involvement, Score 1 (mild) = conjunctival hyperemia, Score 2 (severe) = either ocular-surface epithelial defect or pseudomembrane formation, and Score 3 (very severe) = both ocular-surface epithelial defect and pseudomembrane formation (4). Chronic phase cases were summarized from our multi-center study in Japan (7) and our retrospective analysis for cases seen at Kyoto Prefectural University of Medicine (8)*.

### Treatment Strategy

The use of systemic corticosteroids for acute SJS and TEN is controversial (9–11). Thus, the timing and dose of the administered steroid may be key to obtaining beneficial outcomes.

As a prospective study, we used systemic steroid-pulse and topical betamethasone treatments in five cases diagnosed within 4 days from disease onset (11). All 10 eyes successfully healed without visual dysfunction. In our retrospective analysis, visual prognoses were significantly better in the group receiving topical steroids at the acute stage compared with the no-treatment group (8). In the Japanese treatment guideline, steroid-pulse therapy and topical betamethasone have been recommended in SJS/TEN cases with severe ocular involvement; i.e., an Acute Ocular Severity Score of 2 or 3. In tears obtained from SJS cases at the acute stage with pseudomembrane and epithelial defects, IL-6, IL-8, and MCP-1 were found to be dramatically upregulated (12). Thus, it is important to suppress the ocular surface inflammation. Moreover, initiating treatment with systemic and topical steroids from the onset of the disease appears to be important for the improvement of the visual prognosis.

### Subacute Phase With PED

PEDs occurring in the subacute phase of SJS/TEN are very difficult to treat. Massive inflammation on the ocular surface is often uncontrollable, even with the use of systemic and/or local steroids. Exposure of the corneal stroma can induce infectious or non-infectious corneal stromal thinning and perforation. Long-lasting inflamed PEDs eventually result in symblepharon, as well as conjunctivalization and neovascularization of the cornea, which can lead to blindness.

In such severely inflamed eyes, limbal transplantation and amniotic membrane transplantation (AMT) do not guarantee epithelialization (13–15). Both cultivated corneal limbal epithelial transplantation (CLET) (16, 17) and cultivated oral mucosal epithelial sheet transplantation (COMET) (5) resulted in complete epithelialization of subacute PED in SJS/TEN cases, thus preventing end-stage cicatrization and vision loss. We hypothesize that one of the mechanisms by which COMET has a positive treatment effect on subacute PED is the decrease of massive inflammation on the ocular surface post surgery. While conventional limbal transplantation requires several weeks for the limbal epithelium from the donor cornea to migrate and cover the corneal surface, transplantation of cultivated epithelium covers the entire cornea during surgery and works to resolve the ocular surface inflammation.

### Management at the Chronic Phase

In the chronic stage of SJS/TEN, ocular surface disease arising from the acute stage encompasses a spectrum of ocular manifestations and complications that are often associated with significant visual morbidity (7, 18, 19). Visual impairment and ocular discomfort continue throughout the life of the patient, and usually require long-term medication for optimal control of the disease.

### Ocular Surface Grading Score

To elucidate the profile of chronic ocular-disorder manifestations, we developed an OSGS as an objective method for grading the extent and severity of ocular complications in SJS/TEN (7). Ocular surface findings were classified as corneal complications (i.e., superficial punctate keratopathy, epithelial defect, loss of the palisades of Vogt, conjunctivalization, neovascularization, opacification, and keratinization), conjunctival complications (i.e., hyperemia, and symblepharon formation), and eyelid complications (trichiasis, mucocutaneous junction involvement, meibomian gland involvement, and punctal damage). In the OSGS, these 13 components are graded from 0 to 3.

Among 138 SJS/TEN eyes treated, the most prevalent severe complications were loss of the palisades of Vogt (114 eyes, 82.6%) and meibomian gland involvement (102 eyes, 73.9%). Moreover, visual acuity in 74 of those 138 eyes (53.6 %) was worse than 20/200. Eyes with a higher total score for the three complication categories had poorer vision (*R* = 0.806, *p* < 0.0001). Multivariable regression analysis showed that corneal neovascularization, opacification, keratinization, and cataracts significantly affected logMAR findings (*p* < 0.0001, *p* < 0.0001, *p* = 0.0142, and *p* = 0.0375, respectively) (7).

Recently, our work using OSGS clearly demonstrated the long-term progression of ocular surface cicatrization in chronic-phase SJS/TEN eyes, and in 35 (33.3%) of 105 eyes, the total OSGS worsened during follow-up periods of over 5 years (20). Partial conjunctivalization progressed toward total conjunctivalization, and eyes with total conjunctivalization with partial keratinization progressed toward total keratinization. Thus, strict attention should be paid to eyes with partial conjunctivalization and partial keratinization.

### Management of the Ocular Surface

In the management of chronic SJS/TEN cases, it is important to control ocular surface inflammation. In most cases, topical steroids are considered adequate treatment. However, since steroid-induced glaucoma can develop during the long-term use of topical steroids, strict attention should be paid to secondary glaucoma in SJS/TEN.

Decreasing the number of bacteria on the ocular surface is the key to obtaining complete stabilization of the ocular surface. Thus, appropriate use of topical antibiotics reduces the risk of ocular surface inflammation. Due to the high detection rate of methicillin-resistant *Staphylococcus aureus* (MRSA) and methicillin-resistant *Staphylococcus epidermidis* (MRSA), 0.5% chloramphenicol is often prescribed, and 1% vancomycin eye ointment is known to be effective for MRSA/MRSE conjunctivitis or keratitis (21, 22).

Severe dry eye in SJS/TEN cases is comprised of three important mechanisms: (1) aqueous tear deficiency, (2) decreased wettability of the corneal surface, and (3) increased evaporation. The lacrimal punctum may be closed from scarring or cauterization, which can lead to a high tear meniscus and underestimation of the dry eye severity (23). Thus, it is important to suppress chronic inflammation on the ocular surface. The administration of 2% rebamipide ophthalmic solution reportedly helps to obtain ocular surface stabilization (24–26), and it often reduces or replaces topical steroid use.

### Surgical Interventions

#### AMT

In Japan, AMT was first performed in the mid 1990s (27, 28). Since then, AMT has been performed for recurrent pterygium, severe ocular surface disorders, and ocular surface neoplasia. Amniotic membrane is used after the release of symblepharon as the substrate of epithelial cells. However, in end-stage SJS/TEN cases, the effect of AMT is limited.

#### COMET

In 2002, we started performing COMET for the treatment of severe ocular disorders, including SJS/TEN (29–34). Our retrospective analysis of 86 COMET surgeries revealed that COMET is effective for visual improvement in eyes with chronic SJS/TEN (35). In end-stage cases with severe conjunctivalization, keratinization, and symblepharon, visual improvement was obtained, and the re-constructed ocular surface in those cases was maintained for a long time-period (Figures 1G–I) (36).

Based on the above results, a prospective clinical study of COMET for the treatment of severe ocular surface disorders (i.e., SJS, OCP, and severe thermal/chemical injury) was performed between September 2014 and March 2017 as part of a prospective clinical study under the Advanced Medical Care System in Japan. Thereafter, an investigator-initiated phase 3 clinical trial was started in 2018 to obtain regulatory approval for COMET. In both prospective clinical trials, COMET provided beneficial effects for the release of symblepharon and visual improvement (manuscript in preparation).

Post-surgical management is the key to obtaining favorable results. In our retrospective and prospective clinical studies, systemic corticosteroid (betamethasone, 1 mg/day) and cyclosporine (2–3 mg/kg/day) were administered to prevent postoperative inflammation and immunological response, and then tapered depending on the clinical findings. Nearly all patients required frequent administration of artificial tears, and a therapeutic soft contact lens was used for at least 1-month post surgery to protect the transplanted epithelium from mechanical ablation.

#### Eye Lid Surgery

Trichiasis is a common complication of SJS/TEN at the chronic stage. Double eyelashes and/or entropion are also seen in severely cicatrized cases. Eyelashes touching the cornea induce ocular surface inflammation, and might promote the deterioration of chronic SJS/TEN. Thus, it is optimal to correct these problems, and tarsal wedge resection, gray-line splitting or an autograft of hard-palate are considered depending on the severity of eyelid cicatrization. Interestingly, in a clinically “quiet” chronic SJS/TEN case, folliculitis reportedly existed (37). It should be noted that ocular surface stabilization can be obtained after the successful intervention of these surgeries.

### Visual Rehabilitation Using Limbal-Rigid Contact Lenses

With the aim of reducing the symptoms related to corneal irregularity and dry eye observed in severe OSD cases, we developed a limbal-supported rigid-type CL (Limbal-Rigid CL; Sun Contact Lens Co., Inc., Kyoto, Japan) with a diameter ranging from 13 to 14 mm. When our new CL is worn, a fluid layer exists at the peripheral zone of the lens, and the tears beneath the lens exchange at each blink.

A clinical study of the new Limbal-Rigid CL demonstrated significant improvement in VA and quality of life, particularly in SJS patients (38), and an investigator-initiated study for chronic SJS/TEN cases showed favorable results, the same as in the clinical study (Figures 1J–L) (39). We obtained regulatory approval for the Limbal-Rigid CL in 2016. Moreover, the Limbal-Rigid CL has good wettability, thus reducing eye pain related to severe dryness of the ocular surface, and it provides long-term maintenance of the ocular surface once stabilized.

It should be noted that use of the Limbal Rigid CL greatly enhances the postoperative outcome of COMET. The first step is COMET, followed by initiating Limbal Rigid CL wear (40).

## Summary

Ocular, oral, and nail manifestations are essential for a definitive diagnosis of SJS/TEN with ocular involvement. Acute conjunctivitis followed by skin eruptions with high fever indicates the initial sign of SJS/TEN, and early intervention with systemic and topical steroids at the acute stage appears to be important for the improvement of the visual prognosis. Although it remains impossible to fully restore the ocular surface to its normal healthy state (i.e., that of before disease onset), COMET alone, Limbal Rigid CL use alone, or the combination of both can greatly improve the vision and overall quality of life of patients with chronic-stage SJS/TEN.

## Author Contributions

CS and MU drafted the manuscript. CS, MU, and SK revised the final version of the manuscript. All authors contributed to the article and approved the submitted version.

## Conflict of Interest

The authors declare that the research was conducted in the absence of any commercial or financial relationships that could be construed as a potential conflict of interest.

## Publisher's Note

All claims expressed in this article are solely those of the authors and do not necessarily represent those of their affiliated organizations, or those of the publisher, the editors and the reviewers. Any product that may be evaluated in this article, or claim that may be made by its manufacturer, is not guaranteed or endorsed by the publisher.
